# 

**Published:** 1917-12-29

**Authors:** 


### CONTENTS.

PAGE

**Prospects and Promises** ... ... ... ... ... 261

**Education and Examination** ... ... ... ... 262

**Hospital and Institutional News** ... ... ... 263

A Bombardment Anniversary—Workers' Funds in Country Towns—The Limits of Commercial Advertising—The "Dumping" of Phthisical Cases—Royal Albert Edward Infirmary, Wigan—A Substitute for Christmas Plum-Pudding—A Soldier's Legacy—The Army Council and Food Economy—Sanatorium Treatment—For Extra Services Rendered—Surgical Tuberculosis at Alton—Ministry of Health—A State Medical Service—A Drastic Health By-law—Death in the Shaving-brush—British Red Cross in Holland—Hospital "Cinemas" and Army Idioms—Food and Health—A "Khaki Handicrafts Club" at Bradford—The War-price of Drugs.

**The Worst War of the World** ... ... ... ... 267

V. An Enduring Commemoration. Our Old Army and Our First Defence.

**The Pioneer of Women Practitioners** ... ... 270

Elizabeth Garrett Anderson, M.D. Paris, L.S.A.

**Parliament and the Profession** ... ... ... ... 272

PAGE

**The Matrons' and Sisters' Department** ... 273

The New Year, 1918: What to Read and Study.

Round the Hospitals.

Before Florence Nightingale—A Steep Road and its Endings—Makers of the Modern Hospital—A Good Laugh All Round—Father Christmas To-day and Formerly.

**Once a Ward in Middlesex Hospital** ... ... 27[illegible]

**Matrons' and Sisters' Appointments** ... ... 276

**Metropolitan Hospital Sunday Fund** ... ... 277

Annual Meeting of Constituents: £76,354 Distributed.

**The League of Mercy** ... ... ... ... ... 278

Eighteenth Annual Meeting of Presidents.

**The Book Market** ... ... ... ... ... ... 278

**The Institutional Worker** ... ... (Suppl.) 1

Starting an Auxiliary Military Hospital.

Question for December.

Enquiries and Answers.

Employment and Training

Miscellaneous.

